# Sustained release of actives with Montanide™ ISA 51 VG and Montanide™ ISA 720 VG, two adjuvants dedicated to human therapeutic vaccines

**DOI:** 10.1186/2051-1426-3-S2-P429

**Published:** 2015-11-04

**Authors:** Stephane Ascarateil, Aude Puget, Jerome Gaucheron, Marie-Eve Koziol

**Affiliations:** 1 SEPPIC, Puteaux, France; 2 SEPPIC, Castres, France; 3 SEPPIC Inc, Fairfield, NJ, USA

## Introduction

Montanide™ ISA 51 VG and Montanide™ ISA 720 VG are adjuvants dedicated to human therapeutic vaccines. The adjuvants are rendering stable water-in-oil (W/O) emulsions when mixed with water phase. In these formulations the active ingredients are entrapped in droplets of water surrounded by continuous oil phase. It allows a protection against degradation and slow release of the active into the host. It can bring interesting properties for continuous stimulation of the immune system or to minimize the toxicity of active ingredients.

## In vitro slow release of protein

The proof of concept of the slow release is presented through an in vitro experiment. A model Bovine Serum Albumin (BSA) protein is placed into dialysis bags and the quantity of released protein is measured by ELISA technics. The release of the BSA formulated in W/O emulsions is dramatically delayed as compared to the BSA placed into saline solution.

## In vivo release of caffeine

The effect of several W/O emulsions, based on Montanide™ ISA 720 VG, on the release on a model caffeine active are compared in vivo in rats. Rats were divided into 3 groups containing 4 rats each. The first and second groups were used as controls with intravenous and subcutaneous injections respectively, of 1.5 to 2 ml of aqueous solution containing 40 mg caffeine/kg. The third group received subcutaneously 1.5 to 2 ml of a W/O emulsion containing 40 mg caffeine/kg. The results show that caffeine injected subcutaneously when emulsified is absorbed in a differed way as compared to an aqueous solution caffeine.

## Applications to the release of antibodies

Formulations containing 3% of anti-ovalbumine (OVA) hyper immune sera of mice have been injected to mice. The kinetic apparition of IgG1 has been observed up to 42 days. W/O formulations are inducing a delayed release of IgG1 as compared to non-formulated serum, and are lowering the concentration of circulating antibody. Fransen et al. have emulsified anti CD40 antibodies in Montanide™ ISA 51 VG. The slow release of anti-CD40 could enhance both safety and immune stimulation of the treatment

## Conclusion

Montanide ™ISA 51 VG and Montanide™ ISA 720 VG are vaccine adjuvants already tested in numerous clinical trials. They are rendering W/O emulsions of which slow release properties can be exploited for the administration of monoclonal or polyclonal antibodies. It can be particularly interesting to use these well tolerated vehicles in case of combination treatment for cancer in the view of enhancing the safety of antibodies and associate them to the immune stimulation of the adjuvanted vaccine.

